# Prostate stromal cell proteomics analysis discriminates normal from tumour reactive stromal phenotypes

**DOI:** 10.18632/oncotarget.7716

**Published:** 2016-02-25

**Authors:** Jason P. Webber, Lisa K. Spary, Malcolm D. Mason, Zsuzsanna Tabi, Ian A. Brewis, Aled Clayton

**Affiliations:** ^1^ Division of Cancer and Genetics, School of Medicine, Cardiff University, Cardiff, CF14 4XN, UK; ^2^ Institute of Translation, Innovation, Methodology and Engagement (TIME) Cardiff University School of Medicine Henry Wellcome Building Heath Park, Cardiff, CF14 4XN, UK

**Keywords:** cancer associated fibroblasts, exosomes, angiogenesis, prostate cancer

## Abstract

Changes within interstitial stromal compartments often accompany carcinogenesis, and this is true of prostate cancer. Typically, the tissue becomes populated by myofibroblasts that can promote progression. Not all myofibroblasts exhibit the same negative influence, however, and identifying the aggressive form of myofibroblast may provide useful information at diagnosis. A means of molecularly defining such myofibroblasts is unknown. We compared protein profiles of normal and diseased stroma isolated from prostate cancer patients to identify discriminating hallmarks of disease-associated stroma. We included the stimulation of normal stromal cells with known myofibroblast inducers namely soluble TGFβ and exosome-associated-TGFβ and compared the function and protein profiles arising. In all 6-patients examined, diseased stroma exhibited a pro-angiogenic influence on endothelial cells, generating large multicellular vessel-like structures. Identical structures were apparent following stimulation of normal stroma with exosomes (5/6 patients), but TGFβ-stimulation generated a non-angiogenic stroma. Proteomics highlighted disease-related cytoskeleton alterations such as elevated Transgelin (*TAGLN*). Many of these were also changed following TGFβ or exosome stimulation and did not well discriminate the nature of the stimulus. Soluble TGFβ, however triggered differential expression of proteins related to mitochondrial function including voltage dependent ion channels *VDAC1* and *2*, and this was not found in the other stromal types studied. Surprisingly, Aldehyde Dehydrogenase (*ALDH1A1*), a stem-cell associated protein was detected in normal stromal cells and found to decrease in disease. In summary, we have discovered a set of proteins that contribute to defining disease-associated myofibroblasts, and emphasise the similarity between exosome-generated myofibroblasts and those naturally arising *in situ*.

## INTRODUCTION

Mounting clinical data emphasize the importance of altered stroma as a driver of cancer progression [1–3]. The normal homeostatic function of interstitial tissue becomes disturbed during the early stages of carcinogenesis [4]. This is accompanied by an expanding population of alpha smooth muscle actin (αSMA) positive myofibroblastic cells within the stroma. These myofibroblasts alter the tissue architecture, impacting organ function, and can aid cancer cell proliferation and survival, and support angiogenesis. Ultimately driving the development of high grade tumours associated with poor treatment response [3, 5–7] and poor outcome [4, 8–9].

In xenotransplantation models, myofibroblastic stromal cells taken from cancerous [10], fibrotic or wound-healing tissues [11] can support tumour growth *in vivo*, in part due to enhanced angiogenesis [10]. The capacity of stromal cells to do this, however, is variable [12] and we do not yet understand the nuances of the myofibroblast phenotype which are critical for tumour-promoting effects. Whilst in general terms alterations to cancer associated stroma may indicate poor prognoses [1–3, 13] there are some recent examples in pancreatic cancers where stroma might be protective and inhibit tumour progression [14–15]. This demonstrates functional diversity in the nature of cancer reactive stroma which currently remains poorly understood at a molecular level. Identifying markers of aberrant disease promoting stroma, distinguishing aggressive from more indolent disease, may provide additional information that is useful at diagnosis [16].

The mechanisms initiating myofibroblastic accumulation within the tumour microenvironment centre on complex paracrine factors secreted by tumour cells. These include SDF-1 and TGFβ1 and several others, which may act to recruit cells from other sites into the cancerous tissue, such as bone marrow or adipose tissue derived mesenchymal stem cells [17–18]. Alternatively epithelial or endothelial cells *in situ* may differentiate to a mesenchymal phenotype [19]. Differentiation of resident fibroblasts to myofibroblasts is probably the most extensively studied to date [20] and argued by many to be the likeliest principal source of myofibroblasts. TGFβ can mediate differentiation of fibroblasts into myofibroblasts but this process *in vivo* takes place amongst a host of other factors influencing this process [20]. The manner by which cancer cells dictate the particular type of myofibroblast that arise remains a topic of great interest.

Nanometre sized vesicles, called exosomes, have been proposed as a mechanism by which cancer cells exert control over the cancer microenvironment [21]. This includes induction of myofibroblast differentiation from fibroblasts [22] or from mesenchymal stem cells of bone [23], umbilical cord [24] or adipose-tissue origins [25]. This occurs through vesicular delivery of TGFβ, and likely other factors, that drive stromal precursors towards an apparent disease-promoting myofibroblast [26]. Exactly how representative the stromal response to exosomes is, compared to stromal cells naturally educated *in vivo* by tumour cells, remains unknown.

Our presented study examines the protein repertoire of different forms of stromal cells using a proteomics approach and hypothesises that exosome-stimulation leads to a phenotype with shared features of *in vivo* educated myofibroblasts.

## RESULTS

### Stroma obtained from prostate cancer tissue contains myofibroblasts

**Figure 1:**
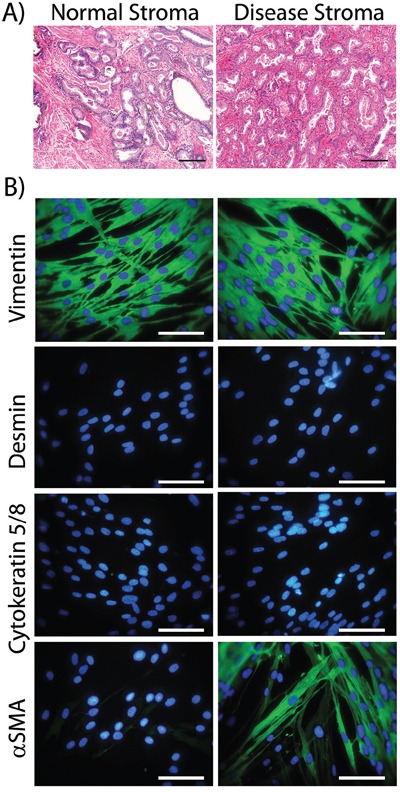
Characterising cultured normal or diseased stromal cells Prostatectomy cores were paraffin embedded, sectioned and stained (H&E) and the histology of tissue from the non-cancerous side of the prostate (Normal Stroma) vs. the cancerous lesion (Disease Stroma) was compared. This is representative of 6 such tissue pairs (Scale Bar=100μm) **A.** Parallel cores were homogenised and used to establish stromal cell cultures. At passage 3 to 5, cells were seeded onto cover slip chamber slides, fixed and indirect immuno-staining was performed for the specified cytoskeleton proteins (Vimentin, Cytokeratin 5 and 8, Desmin or αSMA-green) and DAPI. (Scale Bar=100μm) **B.** Patient WCB1161 is shown and is representative of cultures from 6 patients. The remaining 5 patients are shown in supplemental Fig S1, and the phenotype for 6 patients is summarised in Table 1.

We obtained biopsy material from a total of 6 patients (from the Wales Cancer Bank), in which there was cancer in one half of the prostate and not the other. Histological examination, stained with H&E, of a typical pair of biopsies is shown (Figure 1A), revealing clear differences between the normal and disease tissue. Normal tissue (Figure 1A, left, showing patient WCB1161) demonstrated open glandular structures and a predominantly smooth muscle stromal architecture. This contrasts with disease tissue (Figure 1A, right) in which there was clear hypercellularity and disorganisation of glands, together with an altered, fibrosis-like interstitial stroma and infiltrate. Patient-matched biopsy-pairs were enzymatically homogenised and stromal cultures established as described in the methods.

The phenotype of the cultured cells arising was examined by immuno-fluorescence for a panel of antibodies to discriminate fibroblasts, smooth muscle cells, myofibroblasts and epithelial cells (Figure 1B, showing patient WCB1161). Cells outgrowing from normal tissue exhibited elongated rather than cobblestone morphology, and had the typical appearance of fibroblastic cells. These cells stained strongly positive for the mesenchymal marker Vimentin, but lacked the smooth muscle marker Desmin or the epithelial Cytokeratins. The smooth muscle and myofibroblast marker alpha-smooth muscle actin (αSMA) was absent from normal-tissue derived cultures across all patients. Overall the phenotype here was consistent with a fibroblastic cell type. When compared to morphologically similar cell outgrowths from matched disease tissue, there was no evidence of epithelial or smooth muscle cell (Cytokeratin and Desmin negative) outgrowth. The disease associated cells exhibited a Vimentin and αSMA double positive phenotype; consistent with myofibroblasts. The proportion of αSMA-positive cells in these cultures was variable across the 6 patients and estimations based on manual counting ranged from 35% to 63%. These therefore represent a mixture of fibroblasts and myofibroblasts. The raw data for the remaining 5 patients is shown in supplemental Fig S1 and entirely summarised in Table 1.

**Table 1:** Phenotyping stream cells cultured from paired biopsy tissue

Patient	Normal						Disease		
	Gleason	Vimentin	Cytokeratin	Desmin	αSMA	Vimentin	Cytokeratin	Desmin	αSMA
**WCB949**	3+4	100%	<x1%	0%	0%	100%	0%	0%	38%
**WCB955**	3+4	100%	0%	0%	0%	100%	0%	0%	35%
**WCB1161**	4+4	100%	0%	0%	0%	100%	0%	0%	63%
**WCB1358**	3+4	100%	0%	0%	0%	100%	0%	0%	46%
**WCB1616**	3+4	100%	0%	0%	0%	100%	0%	0%	56%
**WCB1628**	3+4	100%	0%	0%	0%	100%	<1%	0%	61%

Table summarises the pathological assessment of patients biopsy tissue for all 6 patients used in the study, and the immuno-phenotyping analysis of stromal cells isolated from these. The numbers (%) represent an estimation of the proportion of positive cells. Counts were determined manually, from 3 microscopic fields.

### Normal stroma becomes myofibroblastic following stimulation by sTGFβ or cancer exosomes

We next examined the capacity of normal stroma to differentiate into myofibroblasts in response to the classical stimulus of sTGFβ or by treating with exosomes isolated from prostate cancer (Du145) cells. We previously showed exosomes from this source exhibits TGFβ1 tethered to the membrane, at a dose of ∼7.5 pg TGFβ per μg of exosomes. In these experiments therefore a dose of 1.5ng/ml sTGFβ was used to trigger differentiation and this was compared to the equivalent dose of exosomal-TGFβ (200 μ g/ml of exosomes) as described [22]. The emergence of αSMA-positivity was assessed microscopically after three days.

**Figure 2:**
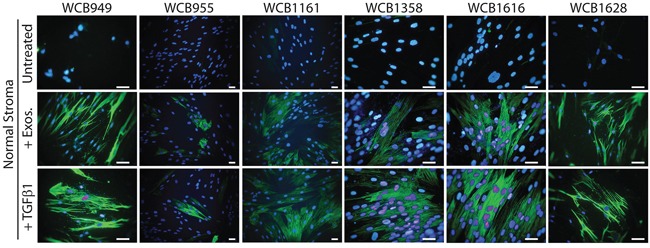
Stimulating normal prostate stromal cells generates myofibroblasts Normal stromal cells were seeded onto glass-chamber slides and growth arrested. After 3 days, media was replaced. For some wells sTGFβ (1.5ng/ml) or Du145 exosomes (200μg/ml) was added and after a further 3 days, the cells were fixed, and stained for αSMA (green) and DAPI. This was performed on normal stroma from all 6 patients as indicated.

There was a clear cut elevation in the proportion of αSMA positive cells in response to sTGFβ as expected, although this was relatively weak for patient WCB955. Elevation of αSMA was present as classical stress fibres along the longitudinal axis of the cell body; typical of a contractile myofibroblastic cell phenotype (Figure 2). A similar response was evident following stimulation with exosomes, and it was not possible to distinguish the stimuli used, based solely on αSMA-expression. Normal prostate stromal cells therefore respond equally well to these stimuli giving rise to a heterogeneous population of fibroblasts and myofibroblasts that are morphologically similar to those naturally occurring in diseased tissue (Figure 2).

### Myofibroblasts exhibit differing capacity for driving angiogenesis

On the basis of αSMA staining alone, it was not possible to discriminate diseased stroma from normal stromal cells that had undergone differentiation in response to our chosen stimuli. We therefore decided to explore the angiogenic potential of the different stromal cell types as previous studies indicate this function may be different across stromal cell types [9, 26].

**Figure 3:**
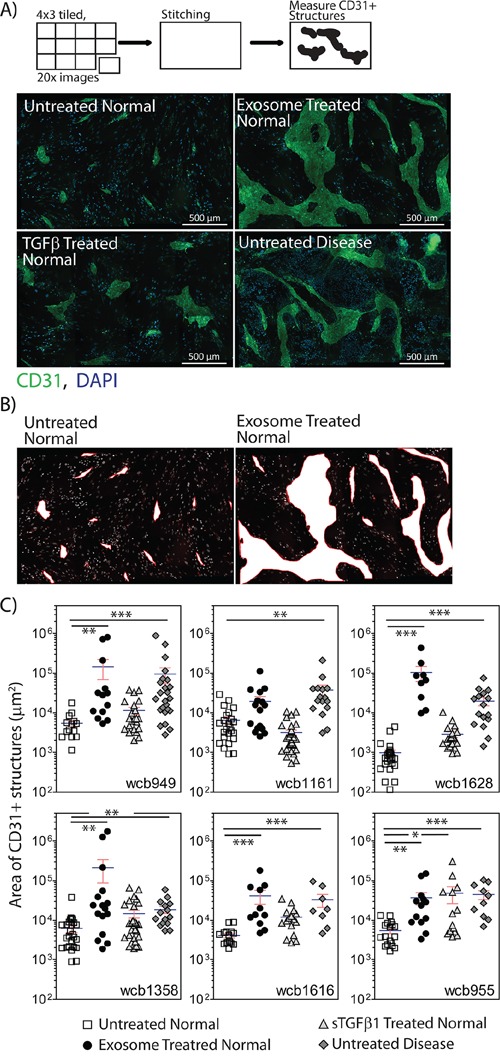
Stromal cells show differential angiogenesis supporting function Monolayers of stromal cells were seeded into 24 well glass-bottomed imaging plates, and were pre-treated as specified, for three days to allow differentiation to myofibroblasts. Endothelial cells (2×10^4^ cell/well in 500μl) were added in a drop-wise and scattered fashion to each well. After 4 days, the cells were fixed and stained for CD31 and DAPI. A series of images was taken in a 4 × 3 grid using a 20x objective, for each duplicate treatment. One composite image is shown for each treatment for the WCB949 patient as a representative example (Scale bar=500μm) **A.** For each image composite, the surface area occupied by each CD31-positive structure was measured. Representative examples of areas taken for such measurements are shown **B.** The CD31-positive area is shown for all treatments in all 6 patients **C.** (*p<0.05, **p<0.01, ***p<0.001, Kruskal-Wallis test with Dunn's multiple comparison post test).

Stromal cells were treated as above, and endothelial cells were added in a scattered/random fashion, to the stromal monolayer. After 6 days, the potential for endothelial cells to proliferate, migrate and organise into vessel-like structures was examined by staining for the endothelial marker CD31. The resultant structures can be large, so multiple 20x images were taken for each condition, and the tiled images were stitched. Each composite image therefore represents approximately ¼ of a 24 well plate well (Figure 3A). Untreated normal stroma did not support the formation of CD31-positive structures, other than the occasional small cell cluster, comprising fewer than 15 cells. In contrast, untreated disease stroma was potent at supporting more elaborate clusters of tens to hundreds of endothelial cells, forming thick and elongated vessel-like structures (Figure 3A). Measuring the area occupied by the CD31-positive structures allowed for a straightforward means of quantifying this pro-angiogenic behaviour. A representation of the typical CD31-positive area measured in shown in Figure 3B. This revealed a significant increase in CD31-positive surface area in response to disease stroma compared to normal stroma in 6 of 6 patients (p<0.001, Figure 3C). Pre-stimulating normal stroma with sTGFβ however gave a poor angiogenic response, with isolated endothelial cell clusters that were small, and not significantly different from normal stroma in 5 of 6 patients (p>0.05). The angiogenic response to exosome-activated normal stroma was very similar to the disease stroma (Figure 3A), where endothelial clusters were large enough to merge, giving a significant elevation in CD31-positive area in 5 of 6 patients (Figure 3C).

From these data, the disease stromal cells and exosome-activated normal stromal cells exhibit similar pro-angiogenic behaviours in these assays. Clearly whilst generating genuine myofibroblasts, sTGFβ treatment appears not to promote a robust angiogenic response, representing a distinct type of myofibroblast.

### Differential protein expression across the stromal cell types

We next examined the protein profile of these stromal cell types, aiming to identify elements that were differentially expressed. We utilised a well established LC-MS proteomics workflow with iTRAQ labelling as we previously described [27–28]. This method provides relative quantity information, and we have used this as a tool to identify proteins of potential interest.

**Figure 4:**
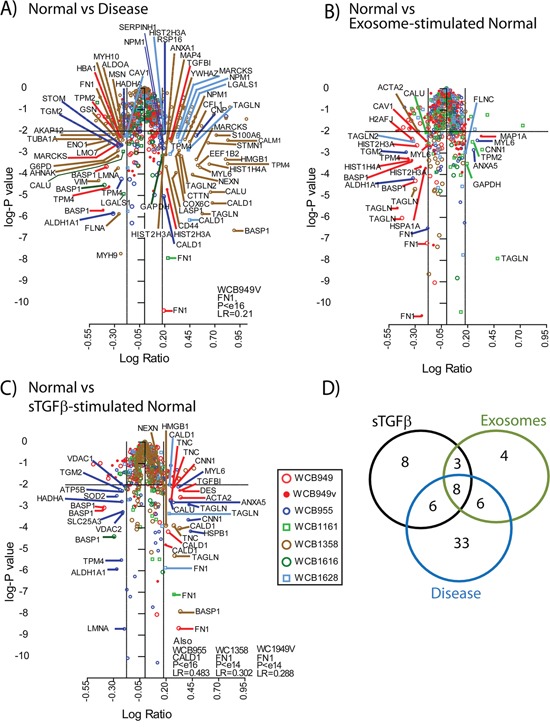
Proteomics analysis of stromal cell types Volcano plots summarising the analysis of differentially expressed proteins, comparing Normal vs Disease stroma **A.** normal vs exosome-stimulated normal stroma **B.** normal vs sTGFβ-stimulated normal stroma **C.,** where coloured symbols represent individual patients (n=6) and WCB949v-depicts the inclusion of data where the protein dose through the workflow was 1, 2, 4 and 8 μg instead of 10ug for all other samples. The thresholds shown indicate a p value <0.01 and a fold change of ±1.5. Identifications outside these criteria were not considered as differentially expressed. A Venn diagram **D.** shows a comparison of the differentially expressed protein lists, and the specific identifications are highlighted in Table 2.

The stromal cells were subjected to whole cell lysis and solubilised proteins were put through a workflow involving trypsin digestion, labelling with isobaric tags and separation by 2D-liquid chromatography. Each specimen was examined freshly and remaining material frozen for the subsequent duplicate run. Comparisons between fresh vs frozen revealed approximately 70% agreement in the identifications (data not shown) and the data for these technical duplicates were merged in the final analysis. The optimal protein loading was assessed using lysates containing 1, 2, 4, 8 or 10 μg, as we have previously observed with other cell types that less protein can give a greater number of protein identifications. This was performed using patient WCB949, and whilst this dose escalation generated some unique proteins, overall there was no real improvement in the numbers of proteins identified. Nevertheless, as these identifications were of robust quality, we have also included these in the analyses (annotated WCB949v accordingly) (in Figure 4 and supplemental Table 1). For remaining samples, these were applied at 10 μg into the workflow.

Differentially expressed proteins were identified by mass-spectrometric analysis, and relative quantity expressed as ratio measurements of treatments compared to untreated normal stroma. The volcano plots (Figure 4) summarise the merged data for all patients (n=6), where coloured symbols represent individual patients, and the plots depict the relative expression data (log ratio), against the significance (log p value). The threshold taken for an identification of interest was based on a fold change of ±1.5 and a p-value <0.01. When comparing disease vs normal stroma we saw the greatest number of differentially regulated proteins. This included 43 that were elevated, such as Calmodulin (*CALM*), Caldesmon (*CALD1*), Transgelins 1 and 2 (*TAGLN*), CD44, Calumenin (*CALU*) and several others. There were another 30 identifications that were downregulated in disease, including Caveolin-1 (*CAV1*), Galectin-1 (*LGALS1*), Tropomyosin alpha-4 (*TPM-4*), Brain acid soluble protein 1(*BASP1*) and others. The proteomics data generated fewer differentially expressed proteins when comparing exosome-stimulated normal stroma to untreated normal stroma (Figure 4B). Here, 8 proteins were elevated, including *TAGLN*, Calponin-1 (*CNN1*) and Annexin A5 (*ANXA5*) and 21 were found decreased including *CAV1*, *BASP1*, and heat shock cognate protein 1A/1B (*HSP71*) (supplemental Table 1). Stimulations with sTGFβ (Figure 4C) revealed a similar signature overall, with 28 elevated proteins including *CALD1*, *TAGLN*, *FN1* and Tenascin (*TENA*). There were 13 downregulated proteins including again *BASP1*, and Tropomyosin alpha-4 (*TPM-4)*. The agreement in differentially expressed proteins across the 6 patients ranged from 0%-26% summarised in supplemental Fig S2.

Using a simple Venn diagram (Figure 4D) we compared the identifications arising across treatment groups. Here we saw a set of 8 proteins that were differentially regulated in all conditions. There was an additional 8 proteins that were unique to the TGFβ-stimulation, but only 4 identifications unique to the exosome-treatment. A total of 33 proteins were distinctive in disease stroma and these data are summarised in Table 2.

**Table 2:** Differentially expressed proteins common and unique according to treatment

8 Proteins common to all treatments	8 Proteins unique to TGFβ-treated stroma	4 Proteins unique to Exosome-treated stroma	33 Proteins unique to disease stroma			
TAGLN	HSPB1	MAP1A	CALM1	LGALS1	RPS16	AHNAK
BASP1	DES	FLNC	S100A6	CTTN	SERPINH1	ENO1
FN1	TNC	HSPA1A	STMN1	COX6C	MSN	GSN
MYL6	VDAC1	H2AFJ	EEF1B2	LASP1	ALDOA	LMO7
CALU	VDAC2		MARCKS	YWHAZ	STOM	AKAP12
TPM4	SLC25A3		CNP	MAP4	MYH9	TUBA1A
TGM2	ATP5B		CFL1	CD44	FLNA	VIM
ALDH1A1	SOD2		NPM1	ANXA1	G6PD	HBA1
			MYH10			

Table specifies the identifications according to the Venn diagram (Figure 4D), emphasising proteins which may discriminate the different types of stroma analysed.

*TAGLN*, Transgelin. *BASP1*, Brain acid soluble protein 1. *FN1*, Fibronectin, *MYL6*, Myosin light polypeptide 6. *CALU*, Calumenin. *TPM4*, Tropomyosin alpha-4 chain. *ALDH1A1*, aldehyde dehydrogenase 1 family member A1. *HSPB1*, Heat shock protein beta-1. *DES*, Desmin. *TNC*, Tenascin-C. *VDAC1*, Voltage-dependent anion-selective channel protein 1. *VDAC2*, Voltage-dependent anion-selective channel protein 2. *SLC25A3*, Phosphate carrier protein, mitochondrial. *ATP5B*, ATP synthase subunit beta, mitochondrial. *SOD2*, Superoxide dismutase [Mn], mitochondrial. *MAP1A*, Microtubule-associated protein 1A. *FLNC*, Filamin-C. *HSPA1A*, Heat shock 70 kDa protein 1A/1B. *H2AFJ*, Histone H2A.J. *CALM1*, Calmodulin. *S100A6*, Protein S100-A6. *STMN1*, Stathmin. *EEF1B2*, Elongation factor 1-beta. *MARCKS*, Myristoylated alanine-rich C-kinase substrate. *CNP*, 2′, 3′-cyclic-nucleotide 3′-phosphodiesterase, *CFL1*, Cofilin-1. *NPM1*, Nucleophosmin. *MYH10*, Myosin-10. LGALS1, Galectin-1. *CTTN*, Src substrate cortactin. *COX6C*, Cytochrome c oxidase subunit 6C. *LASP1*, LIM and SH3 domain protein 1. *YWHAZ*, 14-3-3 protein zeta/delta. *MAP4*, Microtubule-associated protein 4. *CD44*, CD44 antigen. *ANXA1*, Annexin A1. *RPS16*, 40S ribosomal protein S16. *SERPINH1*, Serpin H1. *MSN*, Moesin. *ALDOA*, Fructose-bisphosphate aldolase A. *STOM*, Erythrocyte band 7 integral membrane protein. *MYH9*, Myosin-9. *FLNA*, Filamin-A. *G6PD*, Glucose-6-phosphate 1-dehydrogenase. *AHNAK*, Neuroblast differentiation-associated protein AHNAK. *ENO1*, Alpha-enolase. *GSN*, Gelsolin. *LMO7*, LIM domain only protein 7. *AKAP12*, A-kinase anchor protein 12. *TUBA1A*, Tubulin alpha-1A chain. *VIM*, Vimentin. *HBA1*, Hemoglobin subunit alpha.

### Confirmation of differential protein expression by alternative methods

**Figure 5:**
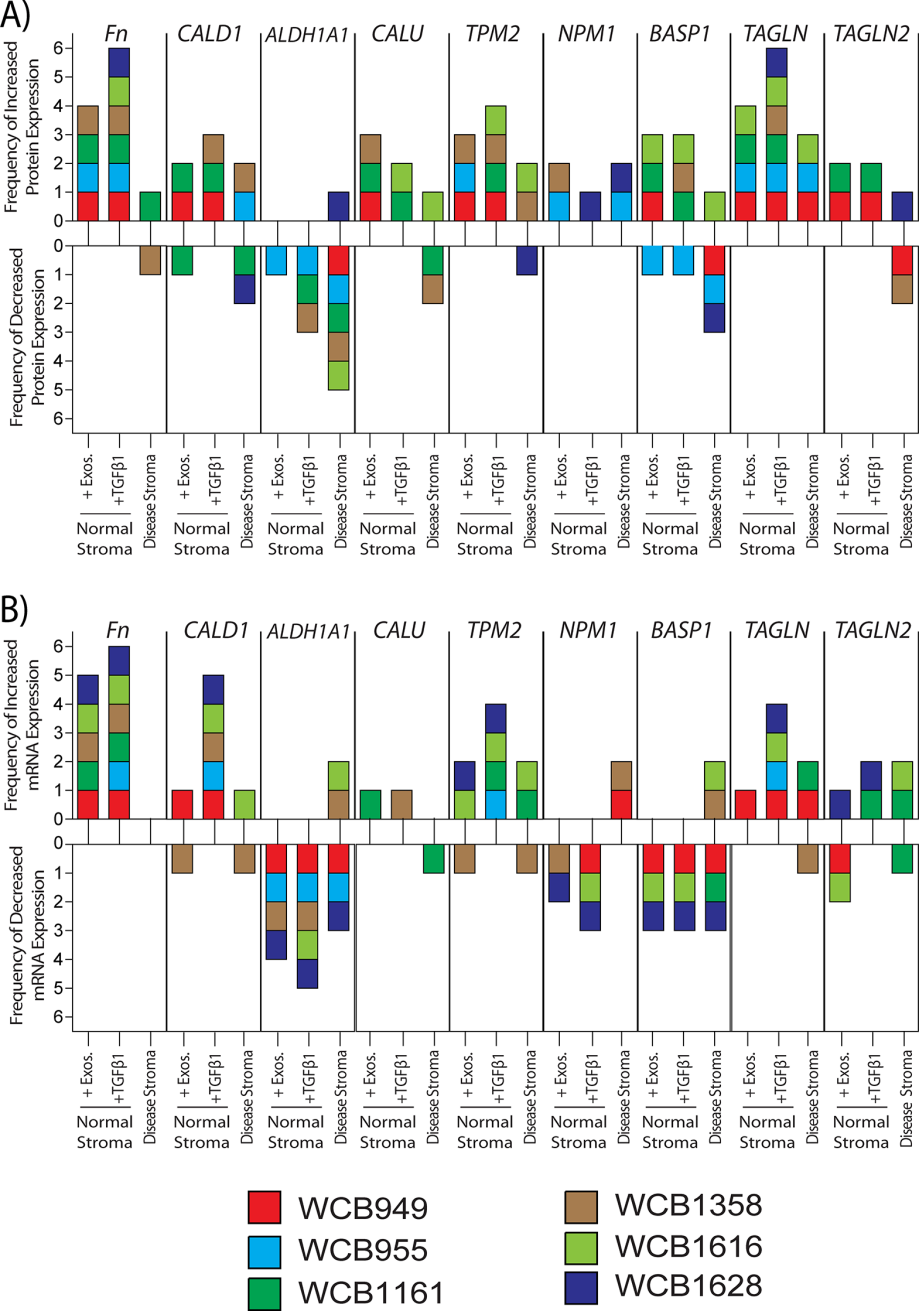
Relative changes in protein and mRNA levels across the treatment groups Western blotting was performed for specified target proteins, and a summary of densitometry analysis is presented. Relative band densities were compared to those of normal stroma, and those showing a fold change of >+1.25 were considered differentially expressed. Changes are shown for individual patients (each represented by coloured boxes), depicting the frequency of positive or negative change **A.** Similarly presented data based on the results of PCR-assays, depicting positive or negative changes in relative mRNA-levels for the same targets **B.** The raw data is shown in supplemental figures S3 and S4 respectively.

From the lists of differentially expressed proteins we selected targets that were identified in more than one patient, with the expectation that these would be verifiable proteins. We performed a series of western blotting and TaqMan™ qPCR-assays to examine differences in expression levels across the four stromal cell types. This was done for all six patients and the results summarised in Figure 5A (protein) and 5B (mRNA), where the individual patients demonstrating either an increase or decrease in expression are shown. For western blots, this was based on band-densitometry and a difference in relative density of >1.25± fold (vs normal stroma) was considered as differentially expressed. The full panel of blots is shown in supplemental Fig S3. Similarly for the TaqMan™-PCR data, target mRNAs were considered differentially expressed if fold change was >±1.25 (vs normal stroma), and of these, all were significantly different (p<0.05). The full panel of relative-expression data is shown in supplemental Fig S4.

With respect to protein, a band for cellular *FN1* was found in all groups regardless of treatment, however in general there was a clear cut elevation in band intensity following short term exposure to sTGFβ or exosomes. This was particularly pronounced for the sTGFβ-treatment in all patients, whereas exosome-stimulus led to a clear elevation in *FN1* in 4/6 patients. Band intensity for *FN1* was more variable for disease stroma, and was only convincing elevated in 1/6 patients, and was decreased in 1/6 patients. Changes therefore in *FN1* were not a consistent feature of disease stroma, but was certainly a marker of recent sTGFβ or exosome-mediated stromal activation (Figure 5A and Fig S3). To some extent a similar pattern was also apparent for *TPM2* where heightened levels were mainly seen in TGFβ1 or exosome stimulated cells (4/6 or 3/6 patients respectively). The pattern of disease related *TPM2* changes was inconsistent with 2/6 patients demonstrating an elevation whilst 1/6 demonstrated decreased expression (Figure 5A and Fig S3). Proteins such as *TAGLN* showed reasonable agreement with the MS data, and elevation was detectable in all 6 TGFβ1 stimulated samples, and a general trend towards elevation in the other samples was apparent.

Proteins such as *CALU*, *CALD1 or BASP1* also showed some contradicting information in western blots, in terms of direction of change across the patients and due to this lack of consistency it was not possible to make any firm conclusions about these targets. Similarly *NPM1* showed hints at elevated expression, but this was seen in only 5 out of a possible 18 samples compared to normal stroma, and is unlikely to be a uniformly altered protein in prostate cancer stroma. Although there were some inconsistencies for *ALDH1A1* the majority of occasions pointed to a decrease in this protein, and particularly so in disease stroma (5/6 patients).

Analysis of relative mRNA levels gave an overall good agreement with the western blot data. *FN1* was elevated following treatment with sTGFβ1 or exosomes in 6/6 and 4/6 samples respectively, but *FN1* mRNA expression, however, was not elevated in disease stroma (Figure 5B and Fig S4). There was increased mRNA expression of both *TPM2* and *TAGLN* in both treated-normal (4/6 patients each) and disease stroma (2/6 patients each), demonstrating good agreement with the western blot data. The pattern of *TAGLN2*, NPM1 and *BASP1* mRNA was again inconsistent, making firm conclusions here difficult. However, also in agreement with the western blot data, *ALDH1A1* mRNA expression decreased following treatment, either with exosomes (4/6 patients) or TGFβ1 (5/6 patients). A reduction in aldehyde dehydrogenase 1 family member A1 (*ALDH1A1)* mRNA was also a feature of diseased stroma in 3/6 patients (Figure 5B and Fig S4).

In conclusion, both the western blot and qPCR data highlight the patient-dependent variation for many of the selected targets, and it is therefore difficult to ascertain a firm profile discriminating disease stroma from the stimulations we have used. Overall, however proteins such as *TPM2, TAGLN* are elevated in diseased stroma, whereas *ALDH1A1* is down regulated allowing potential for normal and diseased stroma to be discriminated.

## DISCUSSION

The onset of myofibroblastic stroma is a general characteristic of tumour-influence, and is in some ways similar to a non-resolving wound-healing response long since described by Dvorak et al [29]. In some settings, such stromal cells support vascularisation and accelerate tumour growth [9–10] and promote invasion and ultimately metastasis through remodelling of the tissue architecture [30]. In contrast recent data in other settings, particularly perhaps in pancreatic cancers, show stromal cells can sometimes exert a protective, tumour attenuating influence [14–15]. Defining molecular features which can predict the functional nature of stromal cells, discriminating those which can promote disease from those which protect, is potentially useful and may provide additional information during histological diagnosis about the aggressivity of the tissue as a whole [31].

In this report we established primary stromal cells from prostatectomy tissue, and explored the proteome of normal and diseased stroma. In agreement with the literature, there was clear evidence of myofibroblastic differentiation occurring under the influence of tumour *in situ*, as the disease-derived cells showed the principal myofibroblast feature, αSMA, yet lacked the smooth muscle marker Desmin. Certainly the diseased stroma showed heterogeneity, with a mixture of fibroblasts and myofibroblasts of varying proportions across the 6 patients tested. Such heterogeneity may be important for tumour promoting function [32] and may well reflect the heterogeneity of the interstitial stroma *in vivo* as reported [4]. Alternatively, the heterogeneity may be an aspect arising due to the culture conditions used. Such myofibroblasts were notably absent in all cultures derived from normal prostate tissue; leading us to believe this is not a culture-artefact and is likely representative of the *in vivo* situation. The ability of the diseased cells to retain this phenotype along serial passaging indicates the tumour-mediated changes are sustained, if not permanent whilst in culture.

Among the aforementioned functional properties ascribed to cancer-associated stroma is their positive influence on angiogenic vessel formation and we found a consistent pro-angiogenic influence with *in vivo*-educated diseased stroma. Even with patients WCB949 and WCB955, where the proportion of myofibroblasts was relatively low (<40%) there was a significant (p<0.001) positive influence on vessel formation. This behaviour was not a property of normal stroma. Short-term stimulation with sTGFβ - the principal cytokine implicated in the generation of myofibroblasts was a potent stimulus for differentiation, but these myofibroblasts remained unable to drive the formation of vessel-like structures in 5/6 patients. Myofibroblasts generated by sTGFβ stimulation of normal stroma have also shown an inability to promote xenograft growth *in vivo* and may in fact exhibit some control of *in vivo* growth [26]. In terms of this function the sTGFβ may represent a protective phenotype, and is at odds with those cells naturally arising at the sites of prostate cancer. Cancer-derived exosomes deliver functionally active TGFβ1 to fibroblasts [22] or to other precursors of myofibroblasts, such as mesenchymal stem cells [23, 25, 33] and potentially mediate formation of a distinct form of myofibroblast [22, 26]. This stimulus consistently drove myofibroblastic differentiation of normal prostate stroma, which in 5/6 patients became potent stimulators of angiogenesis in our vessel-formation assay. This suggests the exosomal-trigger generates myofibroblasts with the functions of diseased stroma, and agrees with our initial premise.

We are not yet sure whether or not the dose of exosomes used here and in these cited studies, is truly representative of the natural dose of vesicles present in tumour interstitial fluid (or in the circulation) of prostate cancer patients, because accurately quantifying vesicles in bio-fluids remains a major challenge. Certainly in studies by other groups, comparable doses of exosomes have been used to drive differentiation [25, 33], and similar doses also used when adding exosomes to *in vivo* model systems [34]. It is also noteworthy that whilst exosomes from prostate cancer can be found in the urine and circulation of patients [35], we show here that tissue from the opposite, histologically normal, side of the prostate remains fibroblastic as opposed to myofibroblastic in nature. This suggests that the diffusion of exosomes across/within the prostate tissue may be somewhat limited, and exosomes do not attain the dose required to form a general organ-wide activation of fibroblasts. Instead, this functional response to exosomes remains a relatively localised phenomenon at least in the specimens of Gleason 6/7 which we have examined.

In terms of the proteomics identifications arising, disease-mediated changes included a set of proteins related to the control of the cytoskeleton such as Transgelin (*TAGLN*), Tropomyosin-4 (*TPM4*), Myosin light chain-6 (*MYL6*) and heavy chain-9 (*MYH9*), Caldesmon (CALD1), Tubulin alpha-1a (*TUBA1A*), Vimentin (*VIM*), A-kinase anchor protein-12 (*AKAP12*), Gelsonin (*GSN*), Filamin A (*FLNA*), Moesin (*MSN*), Cofilin-1 (*CFL1*) and Cortactin (*CTTN*) and these would be consistent with cytoskeleton rearrangement, necessary for acquiring a contractile myofibroblastic phenotype. TAGLN has previously been reported to be upregulated in TGFβ1-induced stromal myofibroblasts, generating a phenotype that resembled reactive stromal cells from patients with prostate cancer [36]. Similarly to MYL6 and TPM4, in our study TAGLN was differentially regulated irrespective of the type of stimulus, and as such these proteins may be general markers of activated stroma. Whilst we identified *CALD1* within the disease stroma and TGFβ1-treated normal stroma by MS, western blot analysis also hinted at an elevation of *CALD1* following exosome-stimulation. Increased *CALD1* expression has been documented as a marker of developing stroma in human foetal prostate xenografts, and may therefore feature in stroma that is dynamically changing [37]. In contrast, proteins like cellular fibronectin (*FN1*) appear principally to be a feature of short-term stimulations in our hands, and sustained elevation was not a consistent feature of diseased stroma. In tissue sections, however enhanced fibronectin expression is a general documented aspect of tumour associated stroma [38]. It is possible that a lack of continual stimulation of stroma by cancer cells in culture explains the normal levels of *FN1* we have seen in the diseased stromal cells; but this is an open question requiring additional investigations.

Other proteins seen altered in all stromal types, compared to normal stroma included Brain acidic soluble protein-1 (*BASP1*) and the Aldehyde dehydrogenase (*ALDH1A1*). *BASP1* is a membrane and cytoskeleton-associated protein predominantly expressed in the neurons of developing brains, and is functionally implicated in neurite outgrowth and motility [39]. This likely involves a function in controlling actin dynamics and membrane structure [40] and hence may be a hitherto undescribed protein involved in the acquisition of myofibroblastic features. Interestingly there is also an inhibitory influence of *BASP1* on the oncogene *MYC* implicating *BASP1* as a tumour suppressor [41]. Its role in the stromal compartment is not to our knowledge known, but potentially *BASP1* modulation may impact a host of *MYC-*dependent processes in the cancer microenvironment. However, there were discrepancies in our data, with western blotting poorly supporting the MS and mRNA-data pointing to down regulated BASP1. We therefore suggest this protein may be of interest in future studies, to ascertain its relationship with cancer-activated stroma. Finding modulated *ALDH1A1* in stroma was similarly unexpected. It's broadly acknowledged as a feature of cancer stem-like cells, related to radioresistance [42] and is a marker that can predict outcome in prostate cancer [43–44]. Whilst the stromal compartment may support a niche for *ALD1A1*-expressing stem cells [45], the importance of stromal *ALDH1A1* is not known. Of interest, the loss of *ALDH1A1* was reported to occur in differentiating myofibroblasts of the cornea, which would agree with our observations here [46], and may point to a general trait related to myofibroblastic differentiation rather than a factor to discriminate bona fide diseased stroma. According to the MS data, CALU was also differentially expressed across the stromal cell types. This extracellular protein, which is implicated in many cellular processes including motility [47] is altered in the stroma of colorectal cancer, and might also be expected as altered in prostate cancer. However, in our model, the changes in CALU, CALD1 and NPM1 were particularly inconsistent across the patients and their relevance in this setting remains ambiguous.

The differentially regulated proteins in disease stroma were not particularly related to a function in angiogenic control except for perhaps C-type natriuretic peptide (*CNP*) which may modulate VEGF levels and is implicated in vessel permeability [48], and annexin-I (*ANXA1*) which has a role in regulating VEGF function [49]. These identifications didn't feature however in the comparably pro-angiogenic exosome-generated myofibroblasts, and may not be essential factors for this functional aspect. There was no identifiable direct angiogenic signature in the data for these pro-angiogenic stromal types. This is probably due to the nature of the LC-MS technology which identifies abundant components in complex mixtures where typically low levels of growth factors and cytokines are simply not detected. It is difficult therefore to speculate on a direct link between these differentially regulated proteins and the observed angiogenic function. A separate study of the stromal secretome would likely be needed for this.

The sTGFβ-generated myofibroblasts uniquely exhibited a striking set of altered mitochondrial proteins including mitochondrial voltage-dependent anion channels (*VDAC1* and *VDAC2*), solute carrier family 25 member 3 (*SLC25A3*), superoxide dismutase-2 (*SOD2*) and ATP synthase subunit 5 (*ATP5*). This is in addition to altered Tenascin-C (*TNC*) a well known matrix component of cancer-reactive and fibrotic stroma [50] indicating such changes are likely valid. Some studies have linked TGFβ with a series of changes to the metabolic status of cancer associated stromal cells, through mitochondrial dysfunction [51], and show such metabolic reprogramming of stroma is requisite for tumour-promoting activity [52]. Such protein changes, however, did not feature in disease-stroma, or exosome-stimulated normal stroma and it is tempting therefore to suggest these changes as aspects of TGFβ stimulation only.

One of the important proteins downregulated in both disease and in exosome-stimulated stroma was Caveolin-1 (*CAV1*). Diminished stromal *CAV1* correlates with increased Gleason score, and reduced relapse-free survival in prostate cancer [53] and therefore marks aggressive disease. Although principally related to endocytosis and other cellular processes *CAV1*, is also a negative regulator of TGFβ1, where loss of *CAV1* boosts TGFβ effects. *CAV1* is a marker that signals cellular autophagy, mitophagy and glycolytic changes are underway, as its loss induces mitochondrial dysfunction [54]. It will be of interest, therefore to examine more directly, potential mitochondrial alterations in stromal cells arising as a consequence of cancer-influence over stroma *in situ*.

In conclusion, utilising functional assays and a proteomics based approach we highlight that exosome-activated normal stromal cells become myofibroblasts akin to those that naturally occurring during disease; with elevation in a set of cytoskeleton related proteins including *TAGLN*. In contrast, we show that the myofibroblast phenotype generated from sTGFβ1 stimulation is distinct, with poor influence on angiogenesis, exhibiting a set of changes in multiple mitochondrial-related components. Finally, our study suggests loss of *ALDH1A1* as a novel marker for disease-related alterations in the stromal compartment, and future studies to better understand the mechanistic importance of ALDH1A1 in controlling the disease related myofibroblast are warranted.

## MATERIALS AND METHODS

### Cell culture

Primary human prostatic stromal cells were generated from ethically obtained tissue collected by the Wales Cancer Bank, from informed and fully consented patients. Cells were isolated from radical retropubic prostatectomy cores, taken from sites of palpable disease and also from apparently normal tissue from the opposite side of the same prostate. Representative cores were stained (H&E), and confirmed by an independent pathologist as cancerous stroma or normal respectively. Homogenized tissue was collagenase I treated (200U/ml; Lonza, Wokingham, UK) and liberated cells plated in stromal cell basal medium (SCBM) (Lonza), which selects for stromal cell types. Cultures were left undisturbed for 7-10 days. At first harvest, cells were subsequently maintained in DMEM:F12 media (Lonza). These cultures were confirmed free of epithelial cells by immuno-fluorescence staining demonstrating lack of cytokeratins, and used in experiments at passage 4-6. Endothelial cells, of human umbilical chord origin, were purchased from Lonza, and maintained in EBM2-media with growth factor supplements (Lonza). For the angiogenesis assay supplements were withdrawn 24h before the experiment, and remained withdrawn for the duration as described [26].

### Exosome isolation

Du145 prostate cancer cells (from ATCC, Teddington, UK), were grown in CELLine bioreactor flasks (Integra Biosciences AG; Hudson, NH, USA) [55] and used as a source of well characterised exosomes for this study [56]. Exosomes were purified from 7-day cell conditioned media using the sucrose cushion method [57] and, as an assessment of purity, all preparations had a particle to protein ratio of >2 × 10^10^ determined by nanoparticle tracking analysis (Nanosight; Malvern Instruments, Worcestershire, UK) and microBCA protein assay (ThermoFisher Scientific, Leicestershire, UK) as described [58].

### Stimulating stromal cells

Stromal cells (normal or diseased) were grown until ∼80% confluent in 10%FBS/DMEM:F12, at which point they were washed three times in DMEM:F12 only, and allowed to growth arrest (under serum starvation) for 72 h. The medium was replaced for normal and diseased stroma. In addition, sTGFβ (1.5ng/ml) or Du145-exosomes (at a matched TGFβ-dose of 1.5ng/ml-TGF=200ug/ml exosomes) was used to stimulate normal stroma. The titration of exosomes to determine this dose was previously performed as described [22-23, 26]. After 72 h, where peak expression of αSMA was previously observed [22] cellular protein (or mRNA) was assessed.

### Vessel formation assay

Stromal cells seeded to 80% confluency in 24 well plates were growth arrested for 3 days then medium replaced with specified stimulus. After a further 3 days, endothelial cells (2 × 10^4^/well) were added in a drop-wise and scattered fashion to randomly distribute them. Cells were left undisturbed for a further 6 days, before fixing for immunofluorescence as described below.

### Immunofluorescent microscopy

Stromal cells were treated as specified then fixed with ice cold acetone : methanol (1:1 ratio) for 5 min. Following solvent evaporation in air, cells were blocked in 1%BSA/PBS for 1h. To evaluate presence of epithelial, smooth muscle cell and fibroblasts/myofibroblasts we used monoclonal antibodies against αSMA, Vimentin, Cytokeratins 8 and 14, and Desmin (Santa Cruz, Dallas, TX, USA). Primary antibodies were used at 1μg/ml, diluted in 0.1% BSA/PBS. After washing, secondary goat anti-mouse IgG Fab'-Alexa 488 conjugate (ThermoFisher Scientific) diluted 1 in 200 in 0.1%BSA/PBS was added for 1h. DAPI (ThermoFisher Scientific) was added for the last 10 min of incubation. After washing, cells were examined by wide field fluorescence microscopy (Axiovert; Zeiss, Cambridge, UK). Cell counts were taken from 3 microscopic fields, to estimate the proportion of αSMA-positive myofibroblasts. For vessel formation assay, endothelial cell-structures were visualised by staining with anti-CD31(Santa Cruz), at 1μg/ml, and a series of 4×3 tiled images taken using a motorised xy stage, and a 20x objective. Tiled images were stitched to form a composite image (using Zen-blue edition 2012 software, Zeiss) and the 2D-area occupied by CD31-positive structures was measured using the Zen-blue software. A composite image was made from each duplicate well per condition and all CD31-positive structures in the image measured. Data are presented as dot-plots (individual measurements) with the mean, for each condition for all 6 patients.

### Preparation of peptides for nano-LC

Stromal cells were washed twice in warm PBS, prior to addition of lysis buffer containing 20nM TEAB, 1% SDS (w/v), 1%NP-40 (v/v) and harvested using a cell scraper at room temperature. After centrifugation to remove insoluble material (5000g/10min), clarified supernatants were subjected to solvent precipitation to remove salts, lipids and detergent (using the 2D clean-up kit;GE Healthcare, Buckinghamshire, UK). The pellets were resuspended in 20 mM TEAB and left overnight at 4°C. The protein content was then determined using the BCA protein assay kit. Samples were then reduced, denatured and alkylated using an iTRAQ labelling kit (Applied Biosystems,) and the standard protocol. The proteins were subjected to digestion with trypsin, 0.8 μg per sample, and incubated at 37°C for 12-16 h. The samples were then dried and resuspended in water with 0.1 % TFA (v/v).

### LC-MALDI and protein identification

Digested peptides (10μg) were separated on a nano-LC system (UltiMate 3000, Dionex, Sunnyvale, USA) using a two-dimensional salt plug method, as previously described [27]. Mass spectrometry was performed using an Applied Biosystems 4800 MALDI TOF/TOF mass spectrometer, as described [27]. The MS/MS data was used to search the latest available Swiss-Prot database (as of 07/2013) using the MASCOT Database search engine v2.1.04 (Matrix Science Ltd, London, UK), embedded into GPS Explorer software v3.6 Build 327 (Applied Biosystems, ThermoFisher Scientific) (default GPS parameters, 1 missed cleavage allowed, fixed modification of MMTS(C), variable modifications of oxidation (M), pyro-glu (N-term E) and pyro-glu (N-term Q), 150 ppm mass tolerance in MS and 0.3 Da mass tolerance for MS/MS which are recommended published tolerances for LC-MALDI [27]. In order for a protein to be identified, there needed to be a minimum of two peptides with MASCOT e-values less than 0.05. There was a false discovery rate (FDR) of 0 % which was determined using the same SwissProt database with the entire sequence randomised. Where more than one protein was identified, the protein with the highest MOWSE score in MASCOT is reported. The analysis was performed with two technical replicates and the data was merged. This was done for a total of 6-patients. For one of the patients, WCB949, the protein amount run through the LC-MALDI was escalated from 1, 2, 5, 8 to 10μg. The data generated here was also included in the analysis and annotated WCB949V (for “variable loading”) in figures and tables.

### MS data analysis

MS-spectra were analysed using ProteinPilot™ (AB-Sciex, Cheshire, UK) running the Paragon™ algorithm [59]. The generated protein and peptide searches were imported into the ProteinPilot™ Descriptive Statistics Template (PSDT), which enabled rapid assessment of the quality of protein identifications and of relative quantity. We report the PSDT output expressing relative quantity estimations as ratiometric data, relative to the normal stroma (normalized to a value of 1). Proteins were considered differentially expressed if the fold change was ≥±1.5 and p-value < 0.01. Data are presented as volcano plots for each treatment condition, and the thresholded data presented as Supplemental Table 1.

### Electrophoresis and immuno-blotting

Stromal cell lysates were generated using RIPA buffer supplemented with 1% protease inhibitors, 1% phenymethylsulfonyl fluoride, and 1% sodium orthovanadate (Santa Cruz). Insoluble material was removed (5000 x g centrifugation) and protein levels determined by Bradford assay (BioRad, Hertfordshire, UK). Protein samples (10μg) were separated through 4-12% NuPAGE™ polyacrylamide gels, with MOPS running buffer (ThermoFisher Scientific). Subsequently, proteins were transferred to PVDF membranes (GE Healthcare), membranes blocked with 5% nonfat powdered milk and 0.1% Tween-20 in PBS for 1 h, and then incubated with primary monoclonal antibody at a concentration of 1-4μg/ml at 4°C overnight. After washes in 0.1% Tween-20/PBS bands were detected using an anti-mouse IgG-horseradish peroxidase conjugated antibody (Santa Cruz) and chemiluminescence substrate (PicoWest, ThermoFisher Scientifc). Primary antibodies included anti-cellular fibronectin (Enzo Life Sciences, Exexter, UK), Caldesmon (ThermoFisher Scientific), Calumenin (Santa Cruz), Tropomyosin-2 (ThermoFisher Scientific), Transgelin-1 (R&D Systems, Abingdon, UK) and Transgelin -2 (Santa Cruz), Nucleophosmin (ThermoFisher Scientific), Aldehyde dehydrogenase (ALDH1A1; R&D Systems), and brain acidic soluble protein-1 (BASP1; ThermoFisher Scientific). Densitometric analysis of western blots was performed using Image J software (NIH, Bethesda, ML, USA). Proteins were considered differentially expressed if relative band density was >±1.25 that observed from the patient-matched untreated normal stroma sample.

### Quantitative (q-PCR)

Extraction of cellular RNA, reverse transcription and PCR was performed as described [22]. The comparative CT method was used for relative quantification of target gene expression against that of a standard reference gene (GAPDH). Data were analyzed using StepOne software (Applied Biosystems, ThermoFisher Scientific). Target mRNAs were considered differentially expressed if fold change was >±1.25 that of the patient-matched untreated normal stroma sample.

### Statistics

Graphs and statistical analyses were performed using Prism-4 software (version 4.03) from Graph Pad, San Diego, CA. In all experiments, with more than two experimental groups, 1-way ANOVA, with Tukey's post test was used. For the vessel-formation assay a Kruskal-Wallis test with Dunn's multiple comparison post test was performed. Differences with p values of 0.05 or less are considered significant *p<0.05, **p<0.001, ***p<0.0001.

## SUPPLEMENTARY FIGURES AND TABLES




