# Supplementary material for: Prostate stromal cell proteomics analysis discriminates normal from tumour reactive stromal phenotypes
Source: Oncotarget. 2016 Feb 25;7(15):20124–39. doi: 10.18632/oncotarget.7716 (PMC4991442; doi:10.18632/oncotarget.7716)
Supplement: Supplementary file 1 [file oncotarget-07-20124-s001.pdf]

SUPPLEMENTARY FIGURES AND TABLE

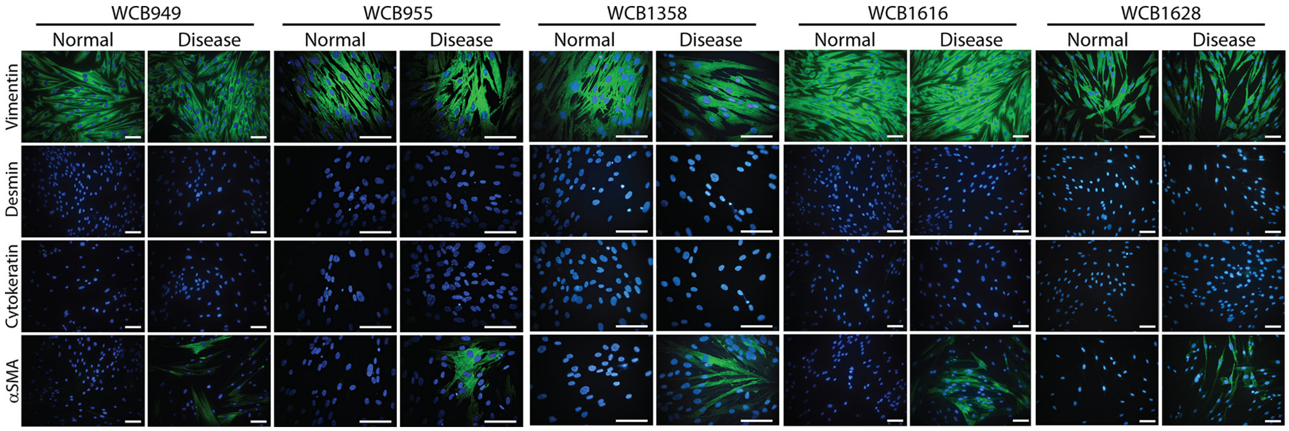

Supplementary Figure S1: Comparing the phenotype of stromal pairs Figure depicts immuno-fluorescent characterisation of primary stromal cells, isolated from normal or matched disease prostate tissue. Included are representative images for 5 patients, with the 6th shown as Figure 1B. Antibodies against Vimentin, Desmin, Cytokeratins 5 and 8 or αSMA (green), and DAPI (blue) (Scale bar=100μm).

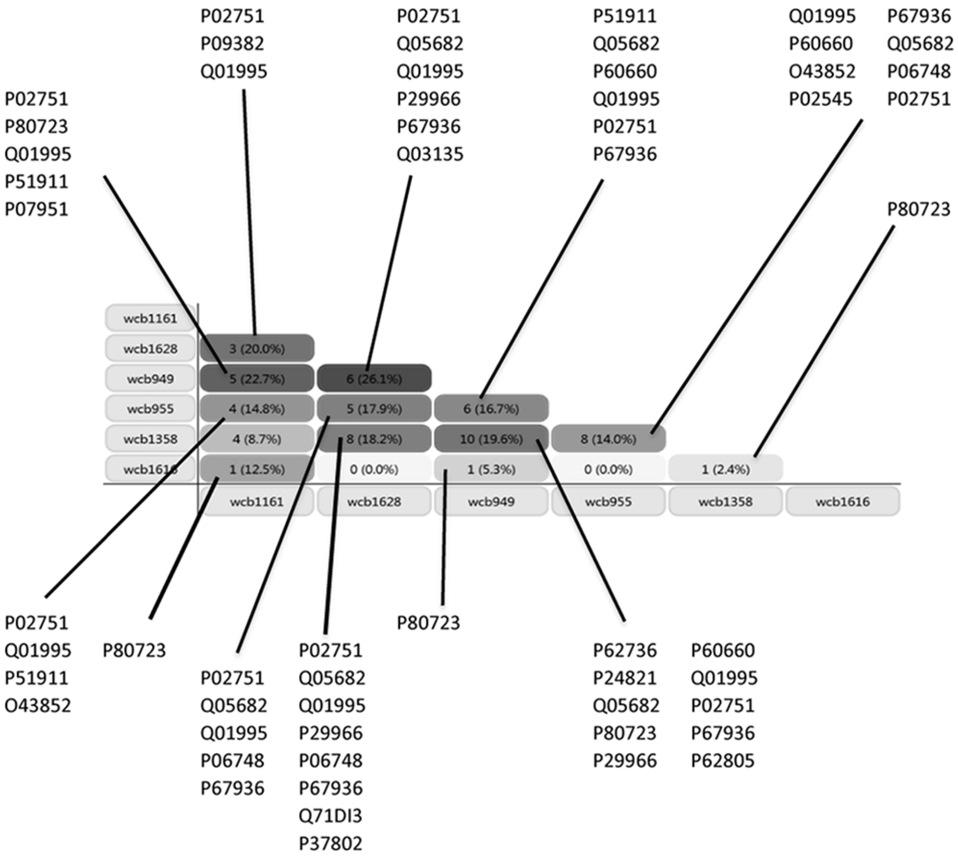

Supplementary Figure S2: Comparing overlap in protein identifications across 6 patients Differentially expressed proteins assigned to each individual patient were compared, using the Venn diagram tool of FunRich. The figure depicts the number of identifications and the proportion of overlap (%) across each of the 6 patients. Uniprot identifiers are given to specify proteins involved, and some of those identified in more than one patient were chosen for verification by other methods.

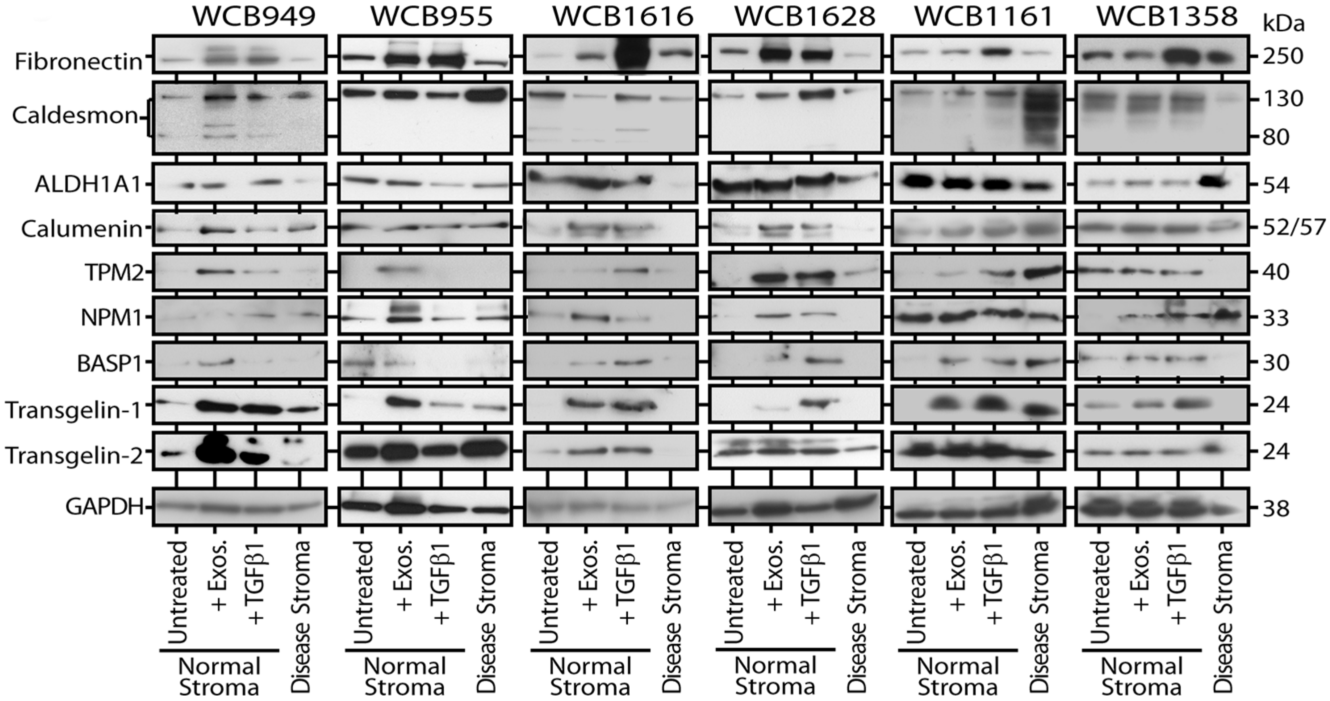

Supplementary Figure S3: Validation of protein targets by western blots analysis. Western blot analysis was used to validate changes in the expression of selected protein targets identified by proteomic analysis. Cell lysates prepared after 72 h stimulation were normalised for total protein, and 10μg loaded per track. The figure shows representative blots of 9 protein targets (plus GAPDH) for each of the 6 patients.

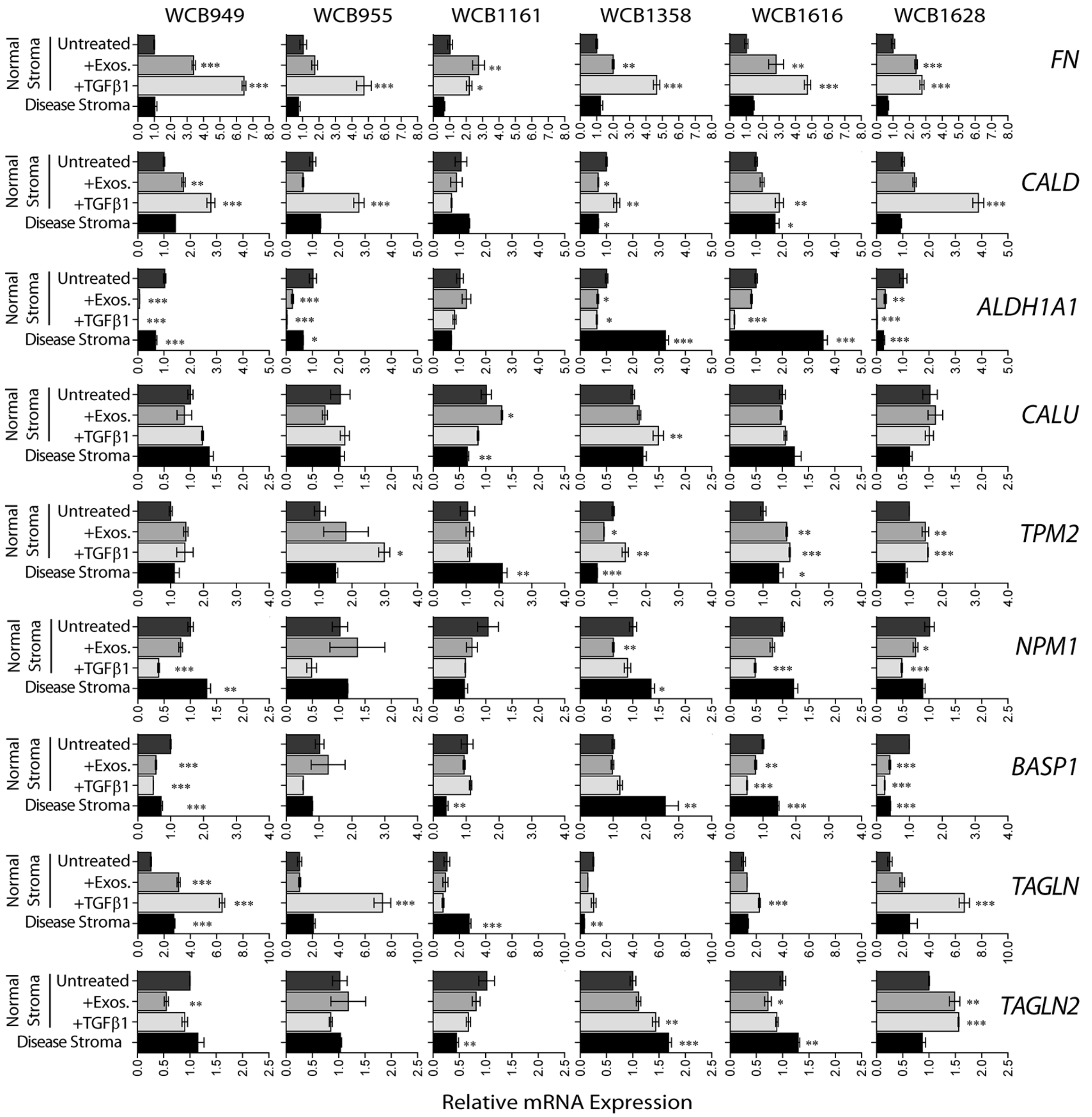

Supplementary Figure S4: Validation of target expression at the transcript level by quantitative PCR. Changes in protein transcript expression were analysed by quantitative PCR. Stromal cell mRNA was isolated at 72 h following treatment (as indicated) and relative transcript level compared to patient-matched untreated normal stromal cells is shown for each of the 6 patient sample sets (mean ±SE of n=3 *p<0.05, **p<0.01, ***p<0.001).

Supplemental Table S1A: Normal Stroma vs Disease

Supplemental Table S1B: Normal Stroma vs Exosome Activated Normal Stroma

Supplemental Table S1C: Normal vs sTGFbeta activated Normal Stroma

See Supplementary File 1
